# Dysregulated sympathovagal interplay contributes to gastric mucosal injury following subarachnoid hemorrhage

**DOI:** 10.3389/fnins.2026.1813675

**Published:** 2026-07-31

**Authors:** Mingli Yao, Meihua Mei, Lingyan Wang, Yufang Wang, Xiangdong Guan, Bin Ouyang

**Affiliations:** Neurosurgical Intensive Care Unit, Department of Critical Care Medicine, The First Affiliated Hospital, Sun Yat-sen University, Guangzhou, China

**Keywords:** autonomic nervous system, gastric mucosal injury, subarachnoid hemorrhage, sympathetic nervous system, vagus nerve

## Abstract

**Background:**

The neural mechanisms underlying brain injury-induced gastric mucosal injury remain poorly defined, with conflicting evidence regarding vagal versus sympathetic dominance. This study aimed to characterize the temporal profile of gastric injury and autonomic nervous activity following subarachnoid hemorrhage (SAH), and to clarify the role of autonomic regulation in SAH-associated gastric pathogenesis.

**Methods:**

SAH was induced in rats by internal carotid artery puncture. Gastric mucosal injury was assessed macroscopically and histopathologically from 6 to 72 h post-SAH. Autonomic activity was evaluated through simultaneous electroneurogram recordings of gastric vagal and sympathetic nerve and measurement of tissue acetylcholine (ACh) and norepinephrine (NE) levels. Functional roles were probed via selective surgical and pharmacological interventions.

**Results:**

SAH induced gastric mucosal erosions/ulcerations within 6 h, peaking at 24 h. This injury was accompanied by elevated tissue levels of ACh (15.90 ± 6.00 vs. 7.86 ± 1.87 ng/mg; *p* = 0.020) and NE (436.76 ± 131.97 vs. 223.66 ± 53.67 pg./mg; *p* = 0.009) at 24 h. By 72 h, autonomic nerve activity was amplified (e.g., vagal positive peak amplitude: 150.50 μV [84.50–360.25] vs. 101.19 μV [47.58–123.45]; *p* = 0.043; sympathetic peak-to-peak amplitude: 267.14 μV [150.50–492.00] vs. 131.00 μV [117.50–149.00]; *p* = 0.023). Both surgical vagotomy and sympathectomy attenuated the injury, reducing the total histopathological score (0.90 [0.60–1.45] and 0.88 [0.58–1.09] vs. 2.15 [1.65–3.77]; *p* = 0.022 and 0.003). Crucially, vagotomy demonstrated superior efficacy: it normalized the macroscopic erosion index (0.00 [0.00–0.00] vs. 13.00 [0.00–16.00]; *p* = 0.038) and significantly reduced tissue levels of both ACh (8.82 ± 1.72 vs. 15.90 ± 6.00 ng/mg; *p* = 0.033) and NE (177.94 ± 37.45 vs. 436.76 ± 131.97 pg./mg; *p* = 0.004), whereas sympathectomy primarily reduced NE (*p* = 0.015).

**Conclusion:**

SAH-induced gastric mucosal injury involves dysregulated sympathovagal interplay, with the vagal pathway potentially playing a predominant role. Further studies are needed to elucidate the mechanisms linking SAH to autonomic dysregulation and consequent gastric mucosal injury.

## 1 Introduction

Stress-related mucosal disease (SRMD) is a common complication in critically ill patients (Barletta et al., 2016; Alhazzani et al., 2012; Bansal et al., 2012), among whom neurocritically ill patients represent a distinct subgroup (Barletta et al., 2018; Ali et al., 2021; Liu et al., 2015). Neurological injury, a recognized risk factor for upper gastrointestinal bleeding, combined with severe physiological stress and critical illness, has been shown to increase the risk of gastrointestinal ulcers and subsequent hemorrhage (Barletta et al., 2018; Schirmer et al., 2012; Liu et al., 2015; Cook and Guyatt, 2018). A 2019 Chinese multicenter study (Wei et al., 2019) reported an overt upper gastrointestinal bleeding incidence of 12.9% in neurocritically ill patients. As the pathogenesis of SRMD in this population remains unclear (Schirmer et al., 2012; Kumaria et al., 2024), current clinical management primarily relies on gastric acid suppression (Bansal et al., 2012). Although effective in reducing gastrointestinal bleeding risk (Liu et al., 2015), proton pump inhibitor prophylaxis may elevate the incidence of adverse events, including hospital-acquired pneumonia and *Clostridium difficile* infection (Rachfalska et al., 2020; Barkun and Bardou, 2018; Barletta et al., 2016; Kumaria et al., 2024). Therefore, elucidating the pathogenesis of SRMD in neurocritically ill patients is clinically imperative.

The pathophysiology of gastric mucosal injury is widely accepted as an imbalance between protective and damaging factors (Schirmer et al., 2012; Kumaria et al., 2024; Cook and Guyatt, 2018). However, the neural regulatory mechanisms underlying SRMD following brain injury and the specific gastric pathophysiology have long remained contentious (Schirmer et al., 2012; Kumaria et al., 2024). This is primarily due to the complex, multilevel neuroanatomical pathways mediating central regulation of gastric function (Levinthal and Strick, 2020; Ray et al., 2020). The autonomic nervous system (ANS) plays a pivotal role in regulating organ functions through brain-to-body signaling, broadly categorized into the sympathetic and parasympathetic nervous systems (Wang et al., 2025a,b). Growing evidence indicates that brain injury-induced autonomic dysfunction is a major cause of associated multi-organ failure (Esterov and Greenwald, 2017; Khalid et al., 2019; Evanson et al., 2025; Wongsripuemtet et al., 2025; Krishnamoorthy et al., 2021).

Under brain injury, the precise alterations in gastric sympathetic and parasympathetic nerve function, and their specific roles in gastric injury, remain unclear (Kumaria et al., 2024). Current evidence conflicts on whether the sympathetic or parasympathetic nervous system is predominantly affected. Regarding the sympathetic nervous system (SNS): substantial evidence indicates that brain injury triggers SNS overactivity via multiple pathways, leading to massive catecholamine release and subsequent dysfunction in peripheral organs (e.g., heart, lung) (Rachfalska et al., 2020; Rizoli et al., 2017; Esterov and Greenwald, 2017; Moussouttas et al., 2015; Salem et al., 2014; Moussouttas et al., 2012; Hasegawa et al., 2022; Khalid et al., 2019; Evanson et al., 2025; Krishnamoorthy et al., 2021). By contrast, research specifically linking SNS overactivity to gastric injury and dysfunction is lacking (Rachfalska et al., 2020). Regarding the parasympathetic nervous system (PSNS): traditional views suggest that brain injury causes vagal overexcitation, resulting in excessive secretion of gastric acid and pepsin, thereby inducing mucosal injury and hemorrhage (Rachfalska et al., 2020; Barletta et al., 2018; Ali et al., 2021; Schirmer et al., 2012; Kumaria et al., 2024). Conversely, emerging evidence indicates that vagotomy exacerbates inflammatory damage in peripheral organs following SAH (He et al., 2013), while vagus nerve stimulation has been shown to ameliorate traumatic brain injury (TBI)-induced systemic inflammation and gastrointestinal dysfunction (Khalid et al., 2019; Bansal et al., 2012). These findings underscore the protective role of the vagus nerve, likely mediated through the cholinergic anti-inflammatory pathway (Kumaria et al., 2024; Silverman et al., 2018). Thus, the role of the PSNS in gastric mucosa injury—whether damaging or protective—remains paradoxically interpreted (Kumaria et al., 2024).

Therefore, the precise role of autonomic dysregulation in the pathogenesis of acute brain injury-induced gastric mucosal injury remains unclear. This study aimed to characterize the temporal profile of gastric mucosal injury following SAH, track corresponding changes in gastric autonomic activity, and elucidate the pathogenic role of autonomic dysregulation.

## 2 Methods and materials

### 2.1 Animals

Male Sprague–Dawley (SD) rats (9–10 weeks old, 300–350 g), specific pathogen-free (SPF) grade, were purchased from the Sun Yat-sen University Laboratory Animal Center [License No. SCXK (Yue) 2021-0029]. Rats were fasted for 12 h prior to anesthesia, with water ad libitum. Aseptic surgeries were performed in the barrier facility of the Animal Experiment Center, Sun Yat-sen University [License No. SYXK (Yue) 2022-0081]. All experimental procedures were reviewed and approved by the Clinical Research and Laboratory Animal Ethics Committee of the First Affiliated Hospital of Sun Yat-sen University (Ethical Approval No. [2021] 857).

### 2.2 Instruments and materials

R520IP small animal anesthesia machine (RWD, Shenzhen, China); surgical microscope (ASX, Shanghai, China); BL-420 N biological signal acquisition and analysis system (TECHMAN, Chengdu, China); Synergy LX multi-functional microplate reader (BioTek, Winooski, VT, United States); KF-PRO-020 automated digital slide scanning system (Jiangfeng Bio, Ningbo, China); and standard microsurgical instruments.

### 2.3 Drugs and reagents

Isoflurane (RWD Life Science, Shenzhen), Penicillin G sodium for injection (1.6 million IU/vial, North China Pharmaceutical Group), 2% Pentobarbital sodium, Atropine sulfate injection (0.5 mg/vial, Tianjin Jinyao Group), Phentolamine mesylate tablets (40 mg/tablet, Beijing Shuguang Pharmaceutical), Propranolol hydrochloride tablets (10 mg/tablet, Yabang Apson Pharmaceutical), 0.9% Physiological saline, Norepinephrine and Acetylcholine ELISA kits (commercially available), Etc.

### 2.4 Model preparation

#### 2.4.1 Grouping

Rats were randomly assigned to the SAH group (sacrificed/evaluated at 6, 12, 24, 48, 72 h post-induction) or the sham-operated group (6, 24, 72 h), with *n* = 8 per group at each time point. Rats that died due to procedure-related complications or were excluded for failing to meet the SAH severity threshold (score <8) were replaced only when the remaining sample size in any experimental subgroup dropped below six. This predefined replacement strategy was applied uniformly across all groups and time points to ensure adequate statistical power.

#### 2.4.2 Anesthesia

For surgical and electrophysiological procedures, anesthesia was induced with 2.5% isoflurane and maintained with 1.5% isoflurane delivered in air at 0.6 L/min; depth was monitored by paw withdrawal and corneal reflexes. For tissue sampling, animals were deeply anesthetized with pentobarbital sodium (100 mg/kg, i.p., 2% solution) and euthanized by cervical dislocation.

#### 2.4.3 SAH modeling

The SAH model was established via microsurgical intracranial internal carotid artery (ICA) puncture, as described by Liu et al. (2017). Animals were positioned; the ventral neck region was shaved and disinfected. A ~ 25 mm midline cervical incision was made. The left common carotid artery (CCA), external carotid artery (ECA), and ICA were carefully exposed via blunt dissection. ECA branches were dissected. The ECA was double-ligated ∼10 mm distal to the carotid bifurcation, then transected with the proximal suture retained for traction. A loose suture was placed ∼5 mm distal to the bifurcation. The CCA and ICA were temporarily occluded with microvascular clips. A partial arteriotomy was made between the loose suture and traction thread on the ECA. A 3-0 monofilament nylon suture was inserted into the ICA through the ECA stump. The ICA clip was removed, and the suture was advanced ~25 mm into the intracranial ICA, and then withdrawn. The loose suture was tightened, and the CCA clip was released. Post-procedural assessment confirmed a palpable ICA pulsation and stable respiration; skin was closed with standard sutures. Sham surgery: The filament was inserted into the ICA but not advanced far enough to puncture the vessel. All other procedures were identical to the SAH group.

Rats received intramuscular penicillin (30,000 IU in 0.75 mL saline), intraperitoneal saline (5 mL), and were placed on a thermostatically controlled heating pad for recovery. Water was provided ad libitum. Food was reintroduced 24 h postoperatively.

### 2.5 Model confirmation and hemorrhage grading

At scheduled time points, rats received a lethal intraperitoneal dose of pentobarbital sodium (100 mg/kg, 2% solution). Following decapitation, skulls were opened along the midline, and the brains were carefully elevated anteriorly to dissect them free from the cranial base.

Hemorrhage severity was scored using Sugawara et al. (2008) grading system. The basal brain surface was divided into six segments. Each segment was assigned a score based on the severity of subarachnoid hemorrhage: 0 = no hemorrhage; 1 = minimal hemorrhage; 2 = moderate hemorrhage with cerebral vessels visible; 3 = dense hemorrhage obscuring the cerebral vessels. The scores from all six segments were summed to calculate a total SAH severity grade. To confirm the successful induction of severe SAH and ensure homogeneity of the experimental group, rats with a total score below 8 were excluded.

### 2.6 Tissue harvesting, measurement, and assessment

#### 2.6.1 Gastric tissue harvesting

Following euthanasia, the abdomens were opened to expose the stomachs. The duodenum (~5 mm distal to the pylorus) and the lower esophagus were ligated. A 10-mL volume of 4% paraformaldehyde (PFA) was injected into the stomach lumen. The esophagus and duodenum were transected distal to the ligatures, and the stomach was removed en bloc. The excised stomach was immersed in 4% PFA at room temperature for 30 min, and then incised along the greater curvature. The mucosal surface was gently cleaned. Gastric mucosal lesions were documented photographically, and the gastric mucosal erosion index (EI) was quantified using microscopy. Representative areas of pathology were selected, fixed, paraffin-embedded, sectioned, and H&E-stained according to standard protocols.

#### 2.6.2 Assessment of gastric mucosal lesions by macroscopic and histopathological analysis

Gastric mucosal lesions were assessed under a microscope at 10 × magnification. The erosion index (EI) was calculated as described previously (Guth et al., 1979). Briefly, the longest diameter (d) of each lesion was measured. Points were assigned for each lesion based on its length: 1 point for d ≤ 1 mm; 2 points for 1 mm < d ≤ 2 mm; 3 points for 2 mm < d ≤ 3 mm, and so on. The point value for any lesion was doubled if its width exceeded 2 mm. The EI for each rat was defined as the sum of the points for all its lesions.

H&E-stained sections were evaluated by blinded observers using a 40x objective lens. Twenty random fields per section were examined. Histopathological damage was scored based on a previously described method (Xu et al., 2018) with minor modifications. The scoring system assessed three parameters: (1) erosion/ulceration; (2) epithelial cell necrosis/desquamation and glandular atrophy/reduction; and (3) hyperemia, hemorrhage, or edema. Each parameter was scored from 0 to 3. For each section, the score for each parameter was averaged across the 20 fields. The total histopathological damage score was defined as the sum of these three average values.

#### 2.6.3 Gastric tissue harvesting and neurotransmitter assay

Following euthanasia by anesthetic overdose, the stomach was rapidly excised. Each stomach was opened along the greater curvature and thoroughly rinsed with ice-cold physiological saline. The glandular stomach was dissected, sectioned into fragments, and blotted on filter paper to remove moisture. The tissue fragments were then transferred to cryovials, snap-frozen in liquid nitrogen, and stored at −80 °C until analysis. Following homogenization, tissue concentrations of NE and ACh were quantified using commercial ELISA kits according to the manufacturers’ instructions.

### 2.7 Monitoring gastric autonomic nerve activity

#### 2.7.1 Nerve isolation

The subdiaphragmatic anterior vagal trunk and the terminal segment of the left greater splanchnic nerve (distal to the adrenal branch, terminating at the celiac ganglion) were carefully dissected free.

#### 2.7.2 Electroneurogram recording and analysis

The isolated nerve segment was gently positioned across the bipolar hook recording electrode. The electrode position and nerve-electrode contact were carefully adjusted to obtain stable and artifact-free signals. The exposed nerve was immersed in warm mineral oil to prevent drying and to provide electrical insulation. A reference electrode was placed on the skin adjacent to the abdominal incision.

Electrophysiological signals were amplified and acquired using a biosignal acquisition and analysis system (BL-420 N, TECHMAN) with the following parameters: sampling rate 20 kHz, low-pass filter 10 kHz, and time constant 0.01 s (equivalent to a high-pass cutoff of ~16 Hz). The low-pass filter was applied to reduce high-frequency noise, and the time constant was set to minimize baseline drift. This methodology was adapted from Silverman et al. (2018).

After signal stabilization, a continuous 5-min recording was obtained. Twenty non-consecutive segments (1–3 s each) were randomly selected from the raw recordings. Segments containing obvious artifacts were visually identified and excluded from analysis. For each segment, spike frequency was determined by threshold-based peak counting, and amplitude (μV) was measured using the built-in interval measurement function of the biosignal analysis system. Mean values of frequency and amplitude were calculated for twenty selected segments. To ensure objectivity, segment selection and analysis were performed by investigators blinded to the experimental groups.

### 2.8 Modulation of gastric autonomic nerve activity

#### 2.8.1 Surgical denervation

Rats were randomly assigned to three groups (*n* = 8 per group): SAH only, SAH + vagotomy, and SAH + sympathectomy. Both denervation procedures were performed immediately after SAH induction. Vagotomy: The subdiaphragmatic anterior and posterior vagal trunks were isolated, and a 2-mm segment was excised from each. The anterior trunk was resected distal to the origin of the hepatic branch, and the posterior trunk was resected at the level of the distal esophagus. Sympathectomy: The left greater splanchnic nerve was isolated. A 2-mm segment was excised distal to the origin of the adrenal branch. All surviving animals were euthanized at the predetermined peak injury time point (24 h post-SAH) for tissue harvesting and analysis.

#### 2.8.2 Pharmacological blockade

Rats were randomly assigned to four groups (*n* = 8 per group): SAH + vehicle, SAH + phentolamine, SAH + propranolol and SAH + atropine. Phentolamine and propranolol are nonselective *α*- and *β*-adrenoceptor antagonists, respectively; atropine is a muscarinic receptor antagonist. Pharmacological interventions were initiated immediately after SAH induction. Atropine (1.0 mg/kg) was administered subcutaneously every 8 h; phentolamine (15 mg/kg) and propranolol (15 mg/kg) were administered by oral gavage every 8 h. The subcutaneous route for atropine was chosen to avoid abrupt cardiovascular effects and to prolong its duration of action. Oral administration of phentolamine and propranolol was selected to mimic clinical practice and to minimize acute hemodynamic fluctuations. The doses were selected based on published rodent studies: atropine (0.1–1.0 mg/kg) reduced indomethacin-induced gastrointestinal mucosal injury (Karádi et al., 1996); phentolamine (5–20 mg/kg) inhibited gastric acid secretion and emptying (Cho et al., 1978); and propranolol (10–45 mg/kg) improved bowel motility (Marcin et al., 2024). The 8-h dosing interval was chosen based on the reported half-lives of these agents (Harrison et al., 1974; Woolfrey et al., 1989; Kerger et al., 1988) to maintain sustained receptor blockade. Vehicle control: equivalent volumes of saline were administered via subcutaneous injection and oral gavage every 8 h. All surviving animals were euthanized at the predetermined peak injury time point for tissue harvesting and analysis.

### 2.9 Statistical analysis

Statistical analyses were performed using IBM SPSS Statistics 26.0 or GraphPad Prism 10.4.2 (San Diego, CA). Continuous data were presented as mean ± standard deviation (SD) or median (interquartile range, IQR). Statistical tests were selected based on data distribution (assessed by the Shapiro–Wilk test) and homogeneity of variance (assessed by Levene’s test). Normally distributed data were analyzed using an unpaired Student’s *t*-test (for two groups) or a one-way ANOVA with Bonferroni *post hoc* test (for more than two groups). Non-normally distributed data were analyzed using the Mann–Whitney U test (for two groups) or the Kruskal-Wallis test with post hoc comparisons corrected by the Bonferroni method (for more than two groups). Statistical significance was defined as *p* < 0.05.

## 3 Results

### 3.1 Study population and sample size

A total of 192 rats were used in two separate cohorts: a time-course cohort (*n* = 136) and an autonomic modulation cohort (*n* = 56). During SAH induction in the time-course cohort, 17 of 94 SAH rats died (18.1%) and six were excluded due to a SAH severity score below 8; no mortality occurred in the sham group (*n* = 42). In the autonomic modulation cohort, six rats died (10.7%) and five were excluded. Overall mortality was 23 of 192 (12.0%), and 158 rats were ultimately included in the final analysis.

### 3.2 Dynamic changes in gastric mucosal injury following SAH

#### 3.2.1 Macroscopic assessment and quantitative analysis of gastric mucosal injury

**Figure 1:**
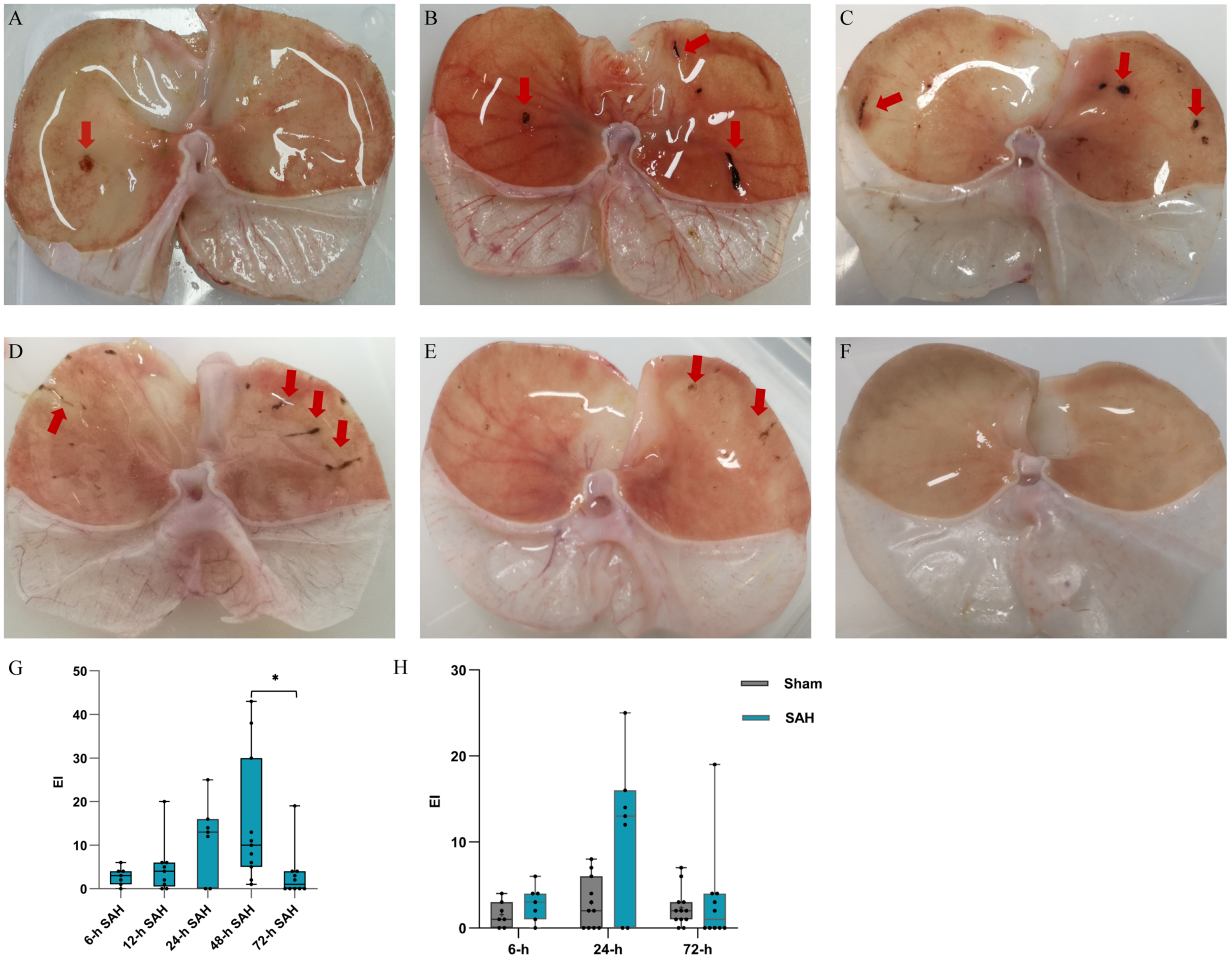
Temporal evolution of gastric mucosal injury following SAH. Representative images: **(A–E)** Macroscopic overview of the gastric mucosa at 6, 12, 24, 48, and 72 h post-SAH, respectively, showing the progression of hemorrhagic lesions. **(F)** Gastric mucosa from sham-operated rats at 24 h, showing an intact surface. Quantitative analysis: **(G)** Erosion index (EI) was significantly reduced at 72 h (*n* = 10) compared to 48 h (*n* = 11) post-SAH (*p* = 0.028). **(H)** EI was elevated in the 24-h SAH group (*n* = 7; median = 13.00 [0.00–16.00]) versus the 24-h sham group (*n* = 11; 2.00 [0.00–6.00]; *p* = 0.058). Arrowheads indicate representative hemorrhagic lesions. * *p* < 0.05, ** *p* < 0.01.

Macroscopic assessment revealed a time-dependent progression of gastric mucosal injury following SAH. At 6 h post-SAH, erosions and ulcerations were observed in the glandular stomach, frequently accompanied by fresh blood (Figure 1A). By 12 and 24 h, the number and severity of lesions increased. These lesions were distributed across both the anterior and posterior walls and along the greater and lesser curvatures, exhibiting diverse morphologies (linear and round) and typically covered with adherent necrotic tissue and hemorrhagic crusts (Figures 1B,C). At 48 h, partial detachment of these crusts was evident in some areas (Figure 1D). By 72 h, crust detachment was more widespread, accompanied by signs of partial healing and a reduction in perilesional mucosal hyperemia compared to earlier time points (Figure 1E). None of these lesions showed evidence of perforation. In contrast, sham-operated rats exhibited normal gastric mucosa with only occasional and sparse lesions (Figure 1F).

The gastric mucosal erosion index (EI) was significantly lower at 72 h than at 48 h post-SAH (median [IQR]: 1.00 [0.00–4.00] vs. 10.00 [5.00–30.00]; *p* = 0.028; Figure 1G). At 24 h, the EI was higher in the SAH group (13.00 [0.00–16.00]) than in the sham group (2.00 [0.00–6.00]), although this difference was not statistically significant (*p* = 0.058; Figure 1H).

#### 3.2.2 Histopathological features and quantitative analysis of gastric mucosal injury

**Figure 2:**
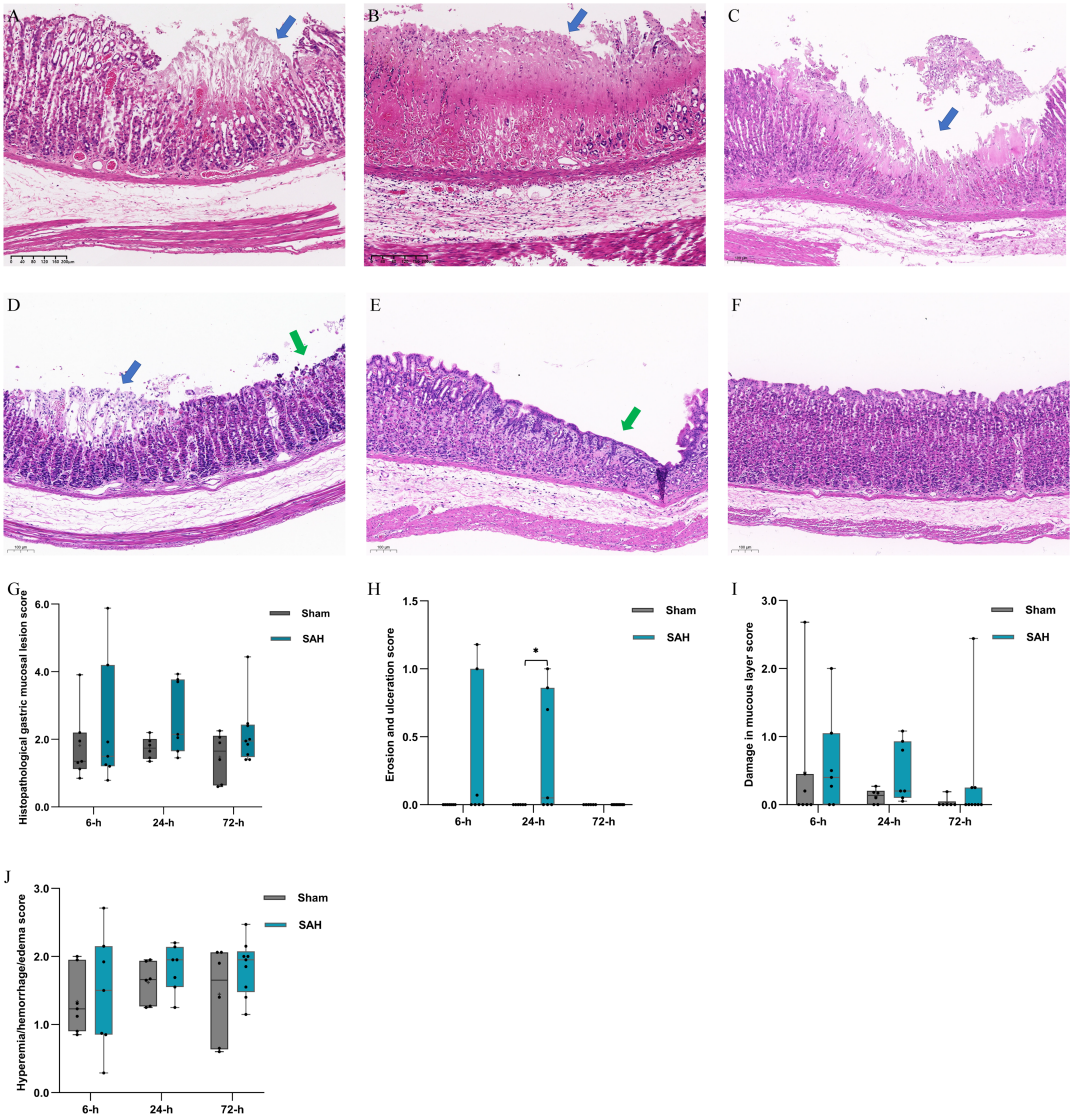
SAH induced time-dependent histopathological changes in the gastric mucosa. Representative H&E-stained sections (10 × objective). Scale bars: **(A,B)** = 200 μm; **(C–F)** = 100 μm. **(A)** 6-h SAH group, showing focal mucosal necrosis with marked congestion, hemorrhage, and edema in the adjacent lamina propria. **(B)** 12-h SAH group, showing necrosis extending through the full mucosal thickness; the adjacent tissue exhibits prominent congestion, hemorrhage, and inflammatory cell infiltration. **(C)** 24-h SAH group, showing sloughing of most necrotic mucosal tissue. **(D)** 48-h SAH group, showing erosions/ulcerations at distinct stages: an early stage with mucosal necrosis and partial sloughing (blue arrow) and a later stage with complete sloughing and initiation of repair (green arrow). **(E)** 72-h SAH group, showing complete sloughing of necrotic tissue with robust cellular proliferation at the base, indicative of ongoing repair. **(F)** 24-h sham group, showing normal gastric mucosal architecture. Quantitative analysis: **(G)** The total histopathological score showed no significant difference between SAH and sham-operated groups at 6, 24, or 72 h. **(H)** The mucosal erosion/ulceration subscore was significantly higher in the 24-h SAH group (*n* = 7) compared with the 24-h sham group (*n* = 6; *p* = 0.036). **(I–J)** The mucosal epithelial injury subscore and the hyperemia/hemorrhage/edema subscore showed no significant differences between SAH and sham‑operated groups at 6, 24, or 72 h. Blue arrows indicate areas of recent mucosal necrosis with partial sloughing. Green arrows indicate areas of complete sloughing with cellular proliferation consistent with repair. * *p* < 0.05, ** *p* < 0.01.

The histopathological evolution of gastric mucosal injury following SAH is summarized in Figure 2. At 6 h post-SAH, foci of mucosal necrosis were observed, accompanied by marked congestion, hemorrhage, and edema in the adjacent lamina propria (Figure 2A). By 12 h, the extent and depth of mucosal necrosis had increased, extending to varying depths and occasionally involving the full mucosal thickness; the muscularis mucosae remained intact. Prominent congestion, hemorrhage, and inflammatory cell infiltration were evident in the mucosa and submucosa adjacent to necrotic foci (Figure 2B). At 24 h post-SAH, most necrotic mucosal tissue had sloughed off (Figure 2C). By 48 h, erosions at varying stages were present: some areas exhibited ongoing mucosal necrosis, while in others, necrotic tissue had completely shed, revealing cellular proliferation at the base indicative of repair (Figure 2D). By 72 h post-SAH, necrotic tissue within lesions had completely sloughed, and cellular proliferation was observed at the base of defects, consistent with ongoing repair (Figure 2E). In contrast, sham-operated rats exhibited normal gastric mucosal architecture without evidence of injury (Figure 2F).

The total histopathological gastric mucosal lesion score did not differ significantly between SAH and sham-operated groups at any time point (Figure 2G). However, the erosion/ulceration subscore was specifically elevated in the 24-h SAH group compared with sham-operated controls (median [IQR]: 0.05 [0.00–0.86] vs. 0.00 [0.00–0.00]; *p* = 0.036; Figure 2H).

### 3.3 Temporal profile of gastric autonomic nerve activity after SAH

#### 3.3.1 Vagal nerve electrophysiological activity

**Figure 3:**
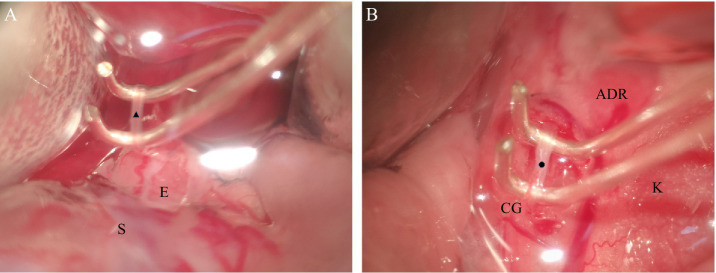
Recording sites for gastric vagal and sympathetic electroneurogram. **(A)** Anterior vagal trunk (subdiaphragmatic). **(B)** Left greater splanchnic nerve (distal to the adrenal branch). Labels: ▲ Anterior vagal trunk (subdiaphragmatic), ● Greater splanchnic nerve (distal segment), S: Stomach, E: Esophagus, CG: Celiac ganglion, ADR: Adrenal gland, K: Kidney.

**Figure 4:**
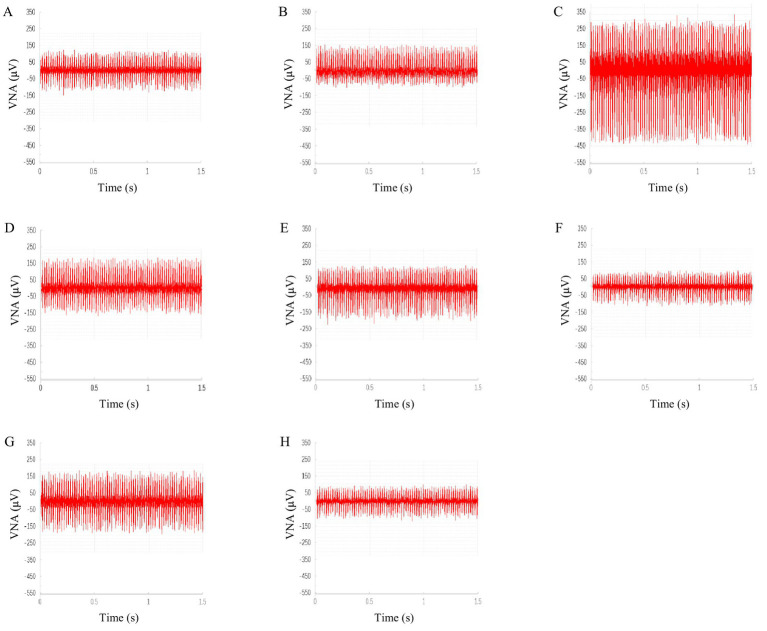
Time course of SAH-induced vagal activity changes. Representative raw electroneurogram traces (1.5-s segments) recorded from the subdiaphragmatic vagal trunk. **(A–E)** Recordings from SAH rats at 6, 12, 24, 48, and 72 h post-procedure, respectively. **(F–H)** Recordings from sham-operated rats at 6, 24, and 72 h post-procedure, respectively. VNA: vagal nerve activity.

**Figure 5:**
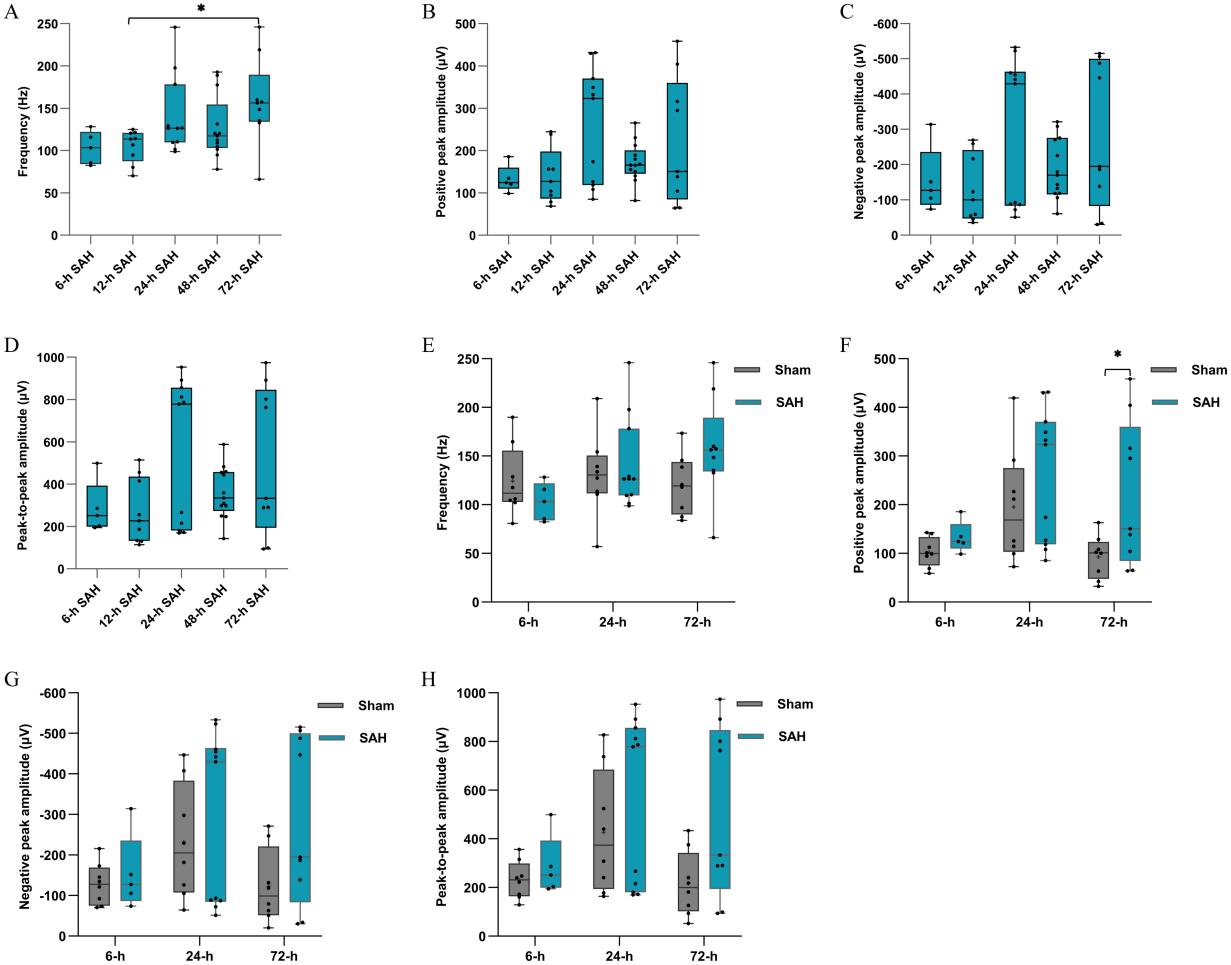
Quantitative analysis of vagal nerve electroneurogram following SAH. **(A–D)** In the SAH group, firing frequency **(A)** and amplitude parameters (**B**: positive; **C**: negative; **D**: peak‑to‑peak) showed increasing trends at 24 and 72 h. The firing frequency **(A)** was significantly higher at 72 h than at 12 h (*n* = 9 per group; *P* = 0.039). **(E–H)** Compared with sham, SAH showed no significant differences in firing frequency **(E)**, negative amplitude **(G)**, or peak‑to‑peak amplitude **(H)**; however, positive peak amplitude **(F)** was significantly increased at 72 h (SAH: *n* = 9; sham: *n* = 8; *P* = 0.043). * *P* < 0.05, ** *P* < 0.01.

The subdiaphragmatic vagal trunk electroneurogram exhibited temporal changes following SAH (Figures 3A, 4A–E). Within the SAH group, firing frequency and all amplitude parameters (positive, negative, and peak-to-peak) showed an increasing trend at 24 and 72 h relative to the other time points (6, 12, and 48 h; Figures 5A–D). The firing frequency at 72 h was significantly higher than at 12 h (median [IQR]: 156.35 Hz [134.05–189.53] vs. 113.34 Hz [87.31–120.86]; *p* = 0.039; Figure 5A). At 72 h, the positive peak amplitude was significantly greater in the SAH group than in the sham-operated group (median [IQR]:150.50 μV [84.50–360.25] vs. 101.19 μV [47.58–123.45]; *p* = 0.043; Figure 5F).

#### 3.3.2 Sympathetic nerve electrophysiological activity

**Figure 6:**
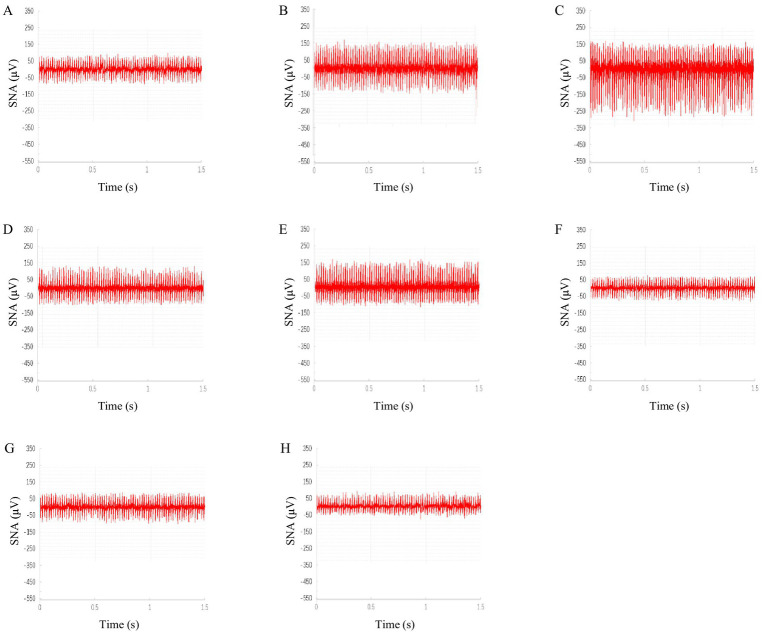
Altered sympathetic nerve activity following SAH. Representative raw electroneurogram traces (1.5-s segments) recorded from the left greater splanchnic nerve (distal segment). **(A–E)** Recordings from SAH rats at 6, 12, 24, 48, and 72 h post-procedure, respectively. **(F–H)** Recordings from sham-operated rats at 6, 24, and 72 h post-procedure, respectively. SNA: sympathetic nerve activity.

**Figure 7:**
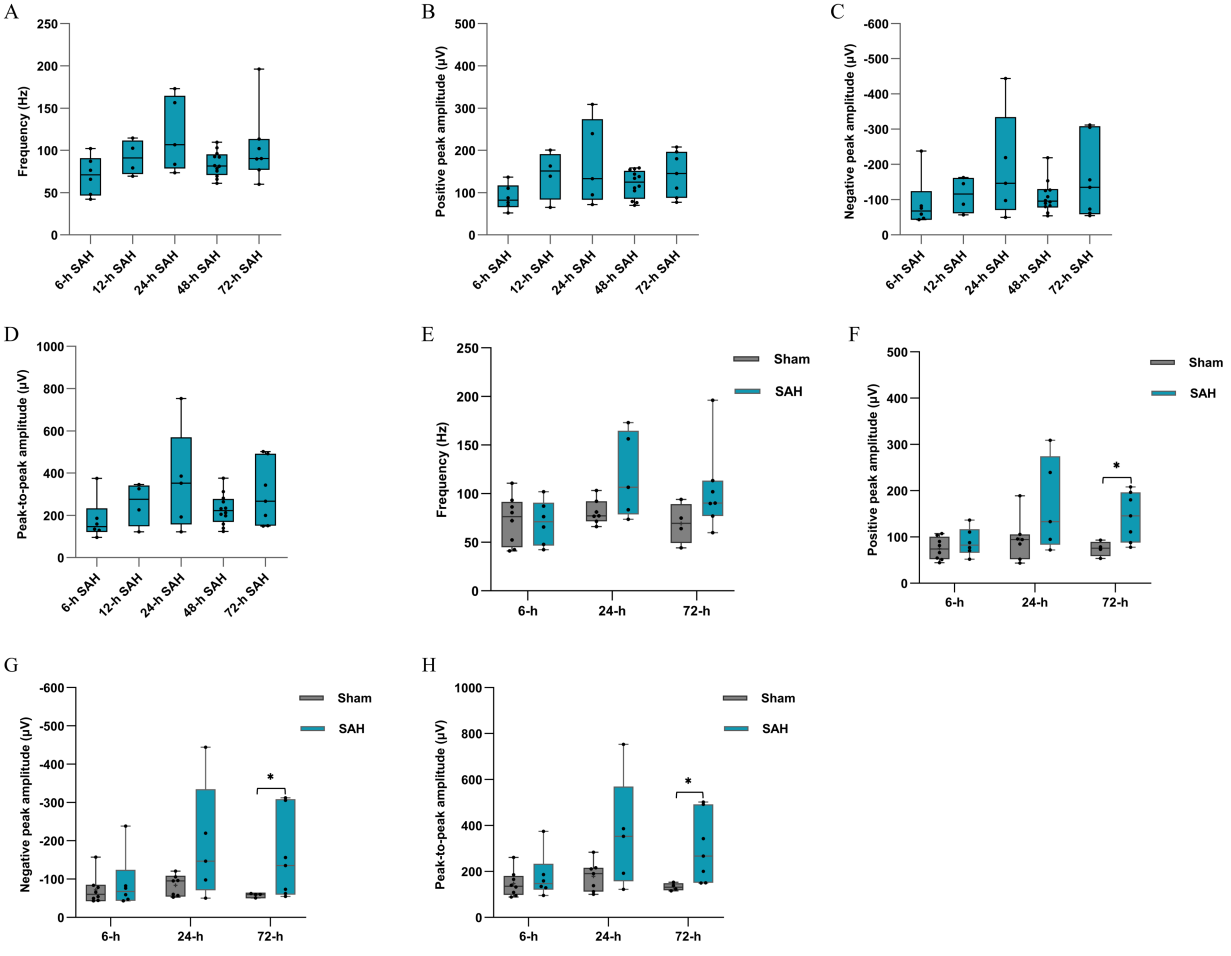
Quantitative analysis of sympathetic nerve electroneurogram following SAH. **(A–D)** In the SAH group, firing frequency **(A)** and amplitude parameters (**B**: positive; **C**: negative; **D**: peak‑to‑peak) showed increasing trends at 24 and 72 h relative to other time points. **(E)** At 24 h, firing frequency showed a non‑significant increasing trend in SAH vs. sham (*P* = 0.088). (**F–H)** At 72 h, SAH showed significantly greater positive (**F**; *P* = 0.038), negative (**G**; *P* = 0.047), and peak‑to‑peak (**H**; *P* = 0.023) amplitudes than sham (SAH, *n* = 7; sham, *n* = 4). * *P* < 0.05, ** *P* < 0.01.

The sympathetic electroneurogram exhibited temporal changes following SAH (Figures 3B, 6A–E). Similarly, firing frequency and amplitude parameters in the SAH group exhibited an increasing trend at 24 and 72 h compared with the remaining time points (6, 12, and 48 h; Figures 7A–D). At 24 h, firing frequency showed a non-significant increasing trend in the SAH versus sham group (median [IQR]: 106.60 Hz [78.58–164.70] vs. 77.17 Hz [71.58–92.18]; *p* = 0.088; Figure 7E). At 72 h, the SAH group exhibited significantly greater values in three amplitude parameters than the sham group: positive peak amplitude (145.50 μV [87.50–196.50] vs. 75.75 μV [58.38–89.38]; *p* = 0.038; Figure 7F), negative peak amplitude (−135.00 μV [−305.50 to −62.00] vs. −59.75 μV [−61.50 to −52.75]; *p* = 0.047; Figure 7G), and peak-to-peak amplitude (267.14 μV [150.50–492.00] vs. 131.00 μV [117.50–149.00]; *p* = 0.023; Figure 7H).

#### 3.3.3 Gastric tissue ACh and NE dynamics

**Figure 8:**
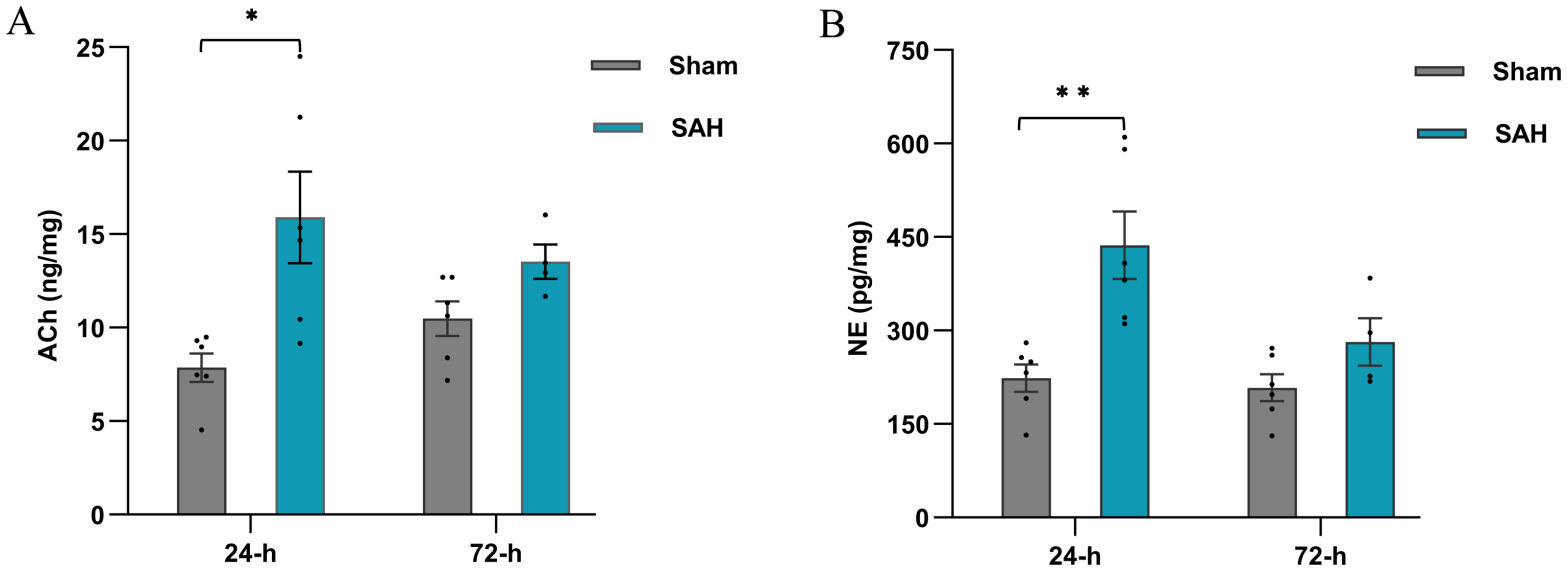
ACh and NE levels in gastric tissue after SAH. **(A)** At 24 h, ACh levels were significantly higher in SAH rats than in sham controls (*n* = 6 per group; *P* = 0.020) and remained elevated at 72 h (SAH, *n* = 4; sham, *n* = 6; *P* = 0.056). **(B)** At 24 h, NE levels were also significantly higher in the SAH group than in the sham group (*n* = 6 per group; *P* = 0.009). Data are presented as mean ± SEM; *P < 0.05, **P < 0.01.

At 24 h post-procedure, gastric tissue levels of both acetylcholine (ACh: mean ± standard deviation: 15.90 ± 6.00 vs. 7.86 ± 1.87 ng/mg; *p* = 0.020; Figure 8A) and norepinephrine (NE: 436.76 ± 131.97 vs. 223.66 ± 53.67 pg./mg; *p* = 0.009; Figure 8B) were significantly elevated in SAH rats compared with sham-operated controls.

By 72 h, ACh levels remained elevated in the SAH group, showing a non-significant trend toward elevation (13.54 ± 1.83 vs. 10.48 ± 2.27 ng/mg; *p* = 0.056; Figure 8A). In contrast, NE levels at this time point were no longer significantly different between groups (281.56 ± 76.85 vs. 207.90 ± 53.03 pg./mg; *p* = 0.108; Figure 8B).

### 3.4 Autonomic neuromodulation attenuated SAH-induced gastric mucosal injury: macroscopic and histopathological evidence

#### 3.4.1 Macroscopic assessment of gastric mucosal injury

**Figure 9:**
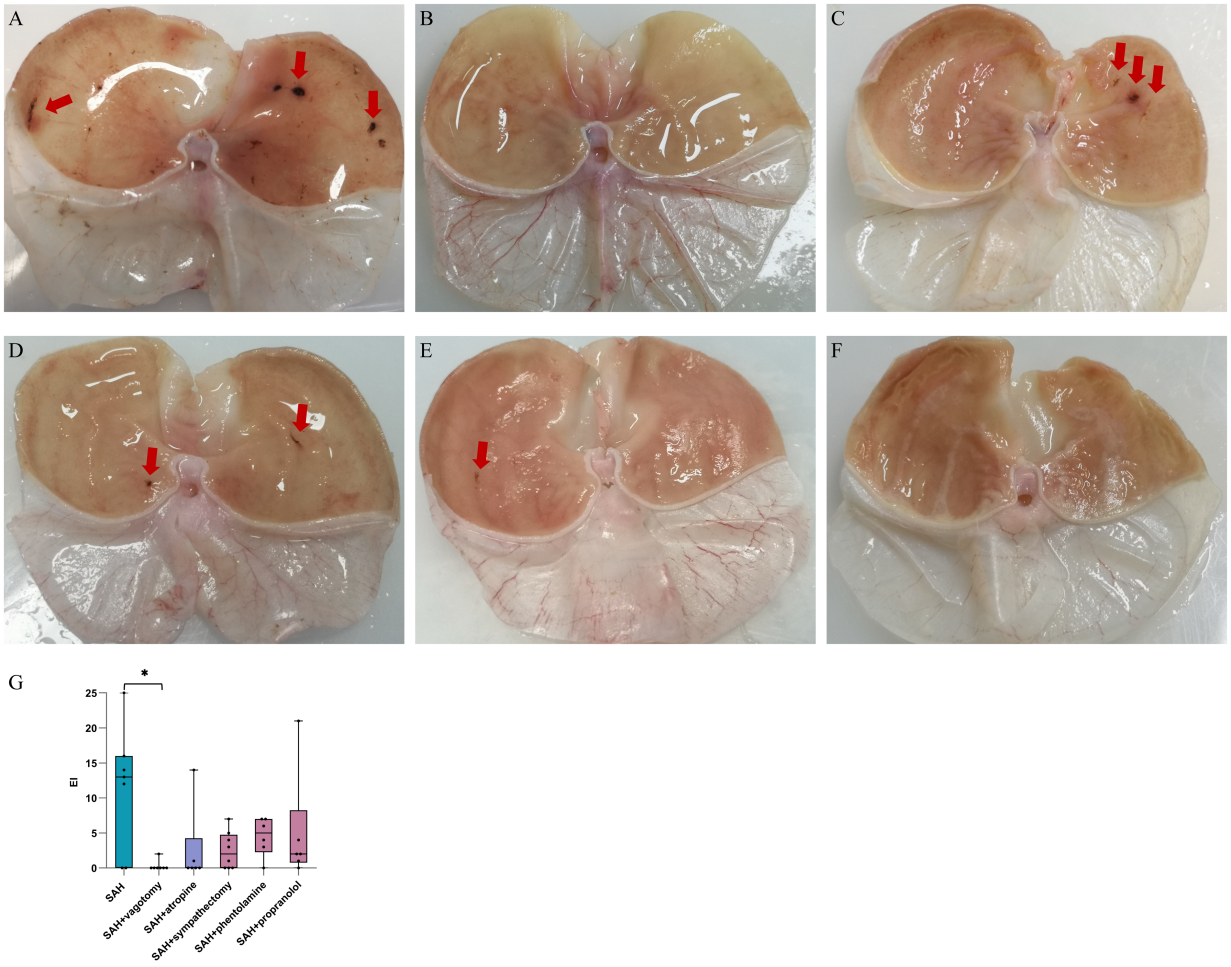
Autonomic neuromodulation attenuated SAH-induced gastric mucosal injury macroscopically. Representative images: **(A)** SAH-alone, showing extensive hemorrhagic erosions and ulcerations. **(B)** SAH + vagotomy, showing an intact surface. **(C)** SAH + atropine, showing a reduction in the number and severity of lesions. **(D)** SAH + sympathectomy, showing sparse lesions. **(E)** SAH + phentolamine, showing sparse lesions. **(F)** SAH + propranolol, with occasional hemorrhagic lesions. Quantitative analysis: **(G)** The gastric mucosal erosion index (EI) was attenuated by autonomic neuromodulation. Vagotomy resulted in a significant reduction in EI compared to the SAH-alone group (*n* = 7 per group; *p* = 0.038). Arrowheads denote representative hemorrhagic lesions. * *p* < 0.05, ** *p* < 0.01.

Autonomic neuromodulation resulted in a macroscopic attenuation of SAH-induced gastric mucosal injury (Figure 9). Quantitative analysis of gastric mucosal lesions showed that vagotomy significantly reduced the EI compared with the SAH-alone group (median [IQR]: 0.00 [0.00–0.00] vs. 13.00 [0.00–16.00]; *p* = 0.038; Figure 9G). In contrast, sympathectomy, atropine, phentolamine, and propranolol interventions showed non-significant EI reductions (Figure 9G).

#### 3.4.2 Histopathological evaluation of gastric mucosal injury

**Figure 10:**
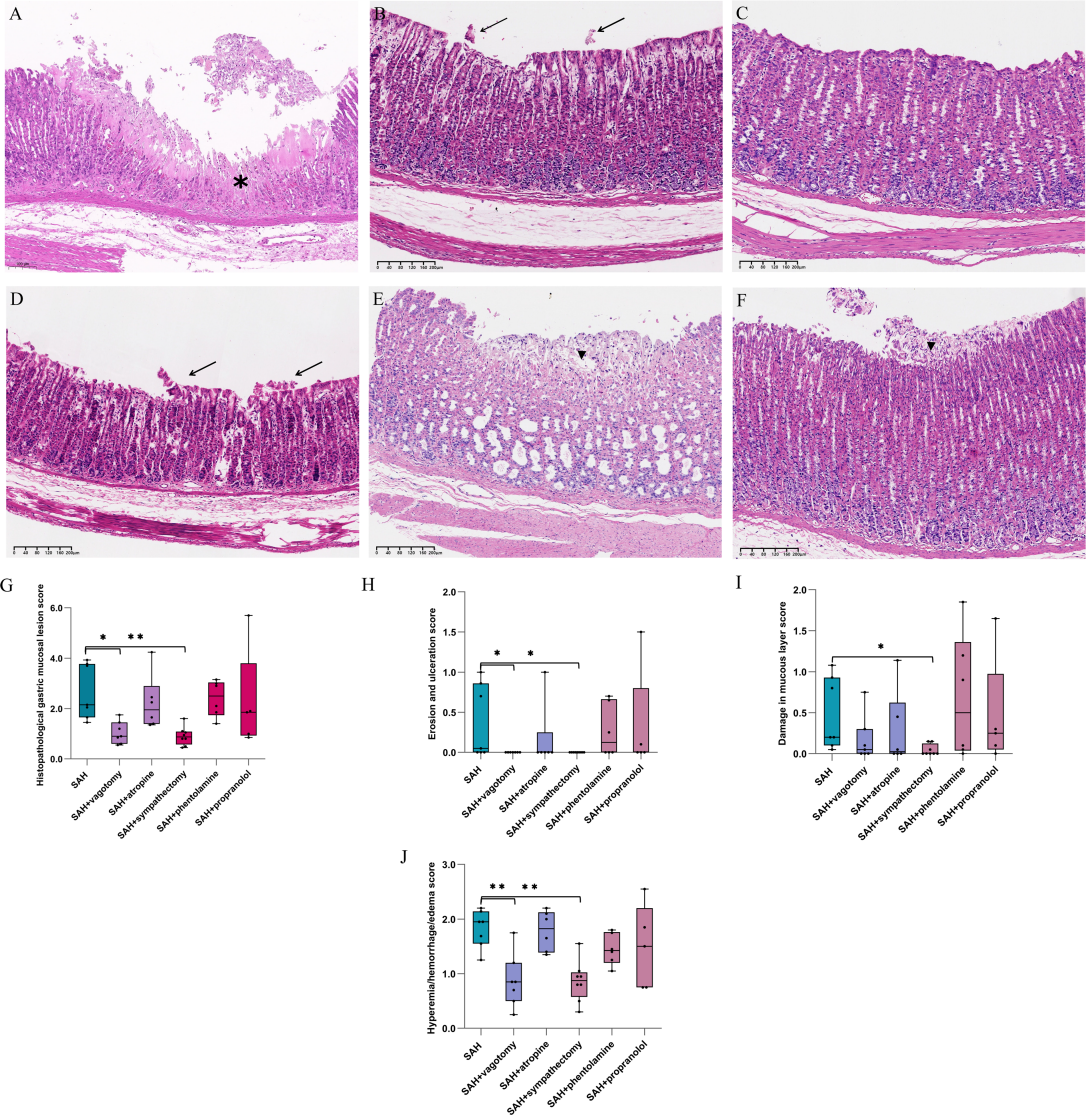
Histopathological assessment of gastric mucosal injury following autonomic neuromodulation. **(A–F)** Representative H&E-stained sections (10 × objective). Scale bars: **(A)** = 100 μm; **(B–F)** = 200 μm. **(A)** SAH-alone group, showing severe injury with deep necrosis (*) and extensive necrotic sloughing. **(B)** SAH + vagotomy and **(C)** SAH + atropine groups exhibited markedly attenuated damage, largely confined to focal epithelial sloughing (→). **(D)** SAH + sympathectomy, **(E)** SAH + phentolamine, and **(F)** SAH + propranolol groups displayed an intermediate phenotype, characterized by superficial necrosis (▼) and moderate epithelial sloughing. **(G–J)** Quantitative analysis of histopathological gastric mucosal lesion scores. **(G)** Compared to SAH-alone controls (*n* = 7), both surgical denervations significantly reduced the total histopathological score: vagotomy (*n* = 7; *p* = 0.022) and sympathectomy (*n* = 8; *p* = 0.003). **(H)** The erosion/ulceration subscore was lower in both the SAH + vagotomy (*p* = 0.021) and SAH + sympathectomy (*p* = 0.016) groups. **(I)** The mucosal epithelial injury subscore was attenuated by sympathectomy (*p* = 0.014). **(J)** The hyperemia/hemorrhage/edema subscore was decreased in both groups (vagotomy, *p* = 0.010; sympathectomy, *p* = 0.009). In contrast, pharmacological blockade with atropine (*n* = 6), phentolamine (*n* = 6), or propranolol (*n* = 5) did not significantly alter any scores. Symbols: →, epithelial sloughing; *, deep necrosis; ▼, superficial necrosis. Data are presented as median with IQR; *p < 0.05, ** *p* < 0.01.

Autonomic neuromodulation attenuated the histopathological severity of SAH-induced gastric mucosal injury. The SAH-alone group exhibited severe pathology, including focal necrosis, extensive necrotic sloughing, and mucosal defects, accompanied by marked hyperemia, hemorrhage, edema, and inflammatory cell infiltration in the adjacent lamina propria (Figure 10A). In contrast, the SAH + vagotomy and SAH + atropine groups showed markedly attenuated injury, characterized only by mild focal epithelial sloughing and an absence of necrosis (Figures 10B,C). Sympathetic-targeted interventions (sympathectomy, phentolamine, and propranolol) demonstrated moderate protection, with histology showing moderate epithelial sloughing and superficial necrosis (Figures 10D–F).

The total histopathological score of gastric mucosal lesions was significantly lower in both the SAH + vagotomy and SAH + sympathectomy groups than in SAH-alone controls (median [IQR]: 0.90 [0.60–1.45] and 0.88 [0.58–1.09] vs. 2.15 [1.65–3.77]; *p* = 0.022 and *p* = 0.003, respectively; Figure 10G). Subscore analysis revealed that erosion/ulceration was reduced by both vagotomy (0.00 [0.00–0.00] vs. 0.05 [0.00–0.86]; *p* = 0.021) and sympathectomy (0.00 [0.00–0.00] vs. 0.05 [0.00–0.86]; *p* = 0.016), as shown in Figure 10H. In contrast, mucosal epithelial injury was attenuated by sympathectomy (0.00 [0.00–0.13] vs. 0.20 [0.10–0.93]; *p* = 0.014) but not significantly by vagotomy (0.05 [0.00–0.30] vs. 0.20 [0.10–0.93]; *p* = 0.164), as shown in Figure 10I. Hyperemia/hemorrhage/ edema subscores decreased significantly following both vagotomy (0.85 [0.50–1.20] vs. 1.95 [1.55–2.14]; *p* = 0.010) and sympathectomy (0.88 [0.58–1.03] vs. 1.95 [1.55–2.14]; *p* = 0.009), as shown in Figure 10J. Pharmacological modulation with atropine, phentolamine, or propranolol, however, showed no significant effect on either the total histopathological score or any subscore compared with SAH-alone controls (Figures 10G–J).

#### 3.4.3 Alterations in gastric NE and ACh levels following autonomic neuromodulation

Both vagotomy and sympathectomy significantly reduced gastric tissue levels of NE compared to the SAH-alone group. In the SAH-alone group, NE levels were 436.76 ± 131.97 pg./mg (*n* = 6). Following vagotomy, levels decreased to 177.94 ± 37.45 pg./mg (*n* = 6; unpaired *t*-test, *p* = 0.004). Sympathectomy reduced NE levels to a median of 192.19 pg./mg (IQR: 181.14–252.50, *n* = 6; Mann–Whitney U test, *p* = 0.015).

In contrast, only vagotomy significantly reduced ACh levels (15.90 ± 6.00 ng/mg in SAH-alone vs. 8.82 ± 1.72 ng/mg after vagotomy; *n* = 6 per group, unpaired *t*-test, *p* = 0.033). Sympathectomy resulted in a non-significant reduction in ACh (median: 9.76 ng/mg, IQR: 9.13–14.63; Mann–Whitney U test, *p* = 0.180).

## 4 Discussion

### 4.1 Manifestations of gastric injury following acute brain injury

Extracranial organ dysfunction is a prevalent complication after acute brain injury (Rachfalska et al., 2020; Mrozek et al., 2020; Robba et al., 2020; Evanson et al., 2025). While cardiac and pulmonary sequelae have been extensively characterized (Rachfalska et al., 2020; Mrozek et al., 2020; Wongsripuemtet et al., 2025; Ziaka and Exadaktylos, 2024), the clinical and pathological features of gastric injury in this context remain poorly defined (Rachfalska et al., 2020; Robba et al., 2020). Cushing’s ulcer—first reported by Harvey Cushing in a postmortem series of 11 patients succumbing shortly after intracranial surgery—is typified by perforated peptic ulcers (Kemp et al., 2015). Morphologically distinct from the diffuse stress-related mucosal lesions seen in general critical illness, these ulcers are typically solitary, deep, and carry a high risk of perforation (Barletta et al., 2018; Kemp et al., 2015; Kumaria et al., 2024). Nevertheless, the precise manifestations of acute brain injury-associated gastric pathology remain uncertain. This uncertainty stems from two constraints: (1) the original autopsy findings were derived from an extreme, small-scale cohort (Kemp et al., 2015), and (2) invasive diagnostic modalities (e.g., gastroscopy) are not routinely employed in clinical practice (Ali et al., 2021).

Using a standardized SAH model, gastric mucosal erosions and ulcerations were observed within 6 h after SAH induction. Pathological severity peaked at 24 h post-SAH, with progressive mucosal restoration observed between 48 and 72 h. These lesions exhibited a multifocal distribution involving the anterior/posterior gastric walls and greater/lesser curvatures. None of these lesions showed evidence of perforation. Histopathological analysis revealed that necrosis was primarily limited to the mucosa, contrasting with transmural involvement reported in human autopsy studies (Kemp et al., 2015). Collectively, these results demonstrate that erosive-ulcerative pathology is the hallmark manifestation of SAH-induced gastric mucosal injury in rats.

### 4.2 Alterations in gastric autonomic regulation after acute brain injury

The stomach, as an autonomic effector organ, receives dual innervation from the sympathetic and parasympathetic nervous systems (Wang et al., 2025a,b; Wehrwein et al., 2016; Levinthal and Strick, 2020). These systems typically exert antagonistic actions on target tissues, where physiological stimuli generally elicit coordinated responses rather than simultaneous activation of both systems (Wehrwein et al., 2016). However, following brain injury, the specific alterations and contributions of sympathetic and parasympathetic signaling to gastric pathology remain unclear (Kumaria et al., 2024). Substantial evidence indicates that brain injury triggers central sympathetic excitation via multiple pathways (Moussouttas et al., 2012; Rabinstein, 2020), culminating in massive circulating catecholamine release (Evanson et al., 2025; Hinson and Sheth, 2012). This triggers systemic SNS hyperactivation, thereby promoting systemic complications in the heart (Salem et al., 2014), lungs (Rabinstein, 2020), kidneys, and other organs (Moussouttas et al., 2015; Moussouttas et al., 2012; Hasegawa et al., 2022; Khalid et al., 2019; Wongsripuemtet et al., 2025; Krishnamoorthy et al., 2021). Beyond this well-documented systemic sympathetic activation, mediated primarily by catecholamine release from the adrenal medulla into the bloodstream, the SNS also operates through distinct local pathways (Schiller et al., 2021). These anatomically and functionally distinct circuits enable organ-specific regulation (Wang et al., 2025a,b). Local sympathetic pathways can be activated independently, releasing norepinephrine directly within the tissue to achieve precise regional control (Wang et al., 2025a,b; Schiller et al., 2021). In contrast, the PSNS operates exclusively via local pathways (Schiller et al., 2021). It provides cholinergic innervation throughout the body, exerting its effects primarily through the release of acetylcholine at neuroeffector junctions (Wang et al., 2025a,b; Wehrwein et al., 2016).

Emerging evidence suggests that the ANS employs multilayered mechanisms to precisely modulate signals for each target organ (Wang et al., 2025a,b). Different anatomical routes underpin the nuanced and organ-specific capabilities of autonomic regulation (Wang et al., 2025a,b; Tao et al., 2021). Specifically, autonomic output to specific end-organs may become activated, suppressed, or remain unchanged relative to others (Esler and Kaye, 2000; Esler, 2011). Accordingly, following brain injury, autonomic outflow may exhibit region-specific alterations. This regional heterogeneity confounds the monitoring of autonomic function. Consequently, global autonomic activity is a poor reflection of local conditions (Esler, 2011), and the state of one organ cannot be generalized to others (Esler and Kaye, 2000). For example, while heart rate variability (HRV) is widely used as a standard method for assessing autonomic nervous functions, it specifically reflects autonomic regulation of sinoatrial node pacemaking and cannot be extrapolated to autonomic activity in non-cardiac organ systems (Hayano and Yuda, 2019).

Therefore, this study combined direct electrophysiological recordings of gastric vagal and sympathetic nerve activity with tissue-level quantification of acetylcholine and norepinephrine to evaluate gastric autonomic function. The results demonstrate that both gastric vagal and sympathetic nerve activity increased following SAH, with peaks occurring at both 24 and 72 h post-ictus.

### 4.3 Autonomic regulation of gastric mucosal injury following acute brain injury

The pathogenesis of brain injury-induced gastric mucosal injury (historically termed Cushing’s ulcer) remains contentious (Kumaria et al., 2024). The traditional hypothesis attributes injury to vagal overactivation resulting from disruption of central autonomic pathways, thereby driving excessive gastric acid and pepsin release (Kemp et al., 2015; Barletta et al., 2018). Conversely, evidence demonstrates that subdiaphragmatic vagotomy exacerbates SAH-induced peripheral organ injury (He et al., 2013); in contrast, vagus nerve stimulation ameliorates TBI-induced systemic inflammation and gastrointestinal dysfunction (Bansal et al., 2012). These findings collectively underscore the vagus nerve’s protective role, which is likely mediated through the cholinergic anti-inflammatory pathway (CAP) (Silverman et al., 2018; Schiller et al., 2021). Thus, conflicting evidence exists regarding the vagus nerve’s net impact on gastric mucosa post-brain injury (detrimental vs. protective) (Kumaria et al., 2024). Regarding sympathetic involvement, robust evidence confirms systemic SNS activation and massive catecholamine release following brain injury, contributing to extracerebral organ dysfunction (Wongsripuemtet et al., 2025; Krishnamoorthy et al., 2021; Rachfalska et al., 2020). However, direct evidence linking SNS excitation to gastric mucosal injury pathogenesis remains lacking (Rachfalska et al., 2020).

This study revealed that SAH activates both sympathetic and parasympathetic gastric nerves. Both sympathectomy and vagotomy attenuated gastric mucosal injury, with vagotomy exhibiting a more pronounced protective effect. Pharmacological blockade, particularly with atropine, showed a trend toward reduced gastric injury severity. These findings indicate that activation of both sympathetic and parasympathetic nerves innervating the stomach contributes to SAH-induced gastric mucosal injury, with parasympathetic pathways playing a predominant role. These observations align with the concept of autonomic plasticity, which involves the flexible recruitment and modulation of autonomic circuits to adapt to physiological and pathological states (Wang et al., 2025a,b).

### 4.4 Sympathovagal interplay in SAH-induced gastric mucosal injury

This study demonstrated increased activity in both vagal and sympathetic nerves innervating the stomach in SAH rats. Subdiaphragmatic vagotomy significantly reduced gastric tissue levels of both acetylcholine and norepinephrine. Sympathectomy markedly decreased norepinephrine levels and resulted in a non-significant decrease in acetylcholine. Both vagotomy and sympathectomy attenuated gastric mucosal injury. These findings indicate that bidirectional interactions between sympathetic and parasympathetic pathways mediate SAH-induced gastric mucosal injury. This is consistent with growing evidence for extensive intra- and inter-system communication within the ANS (Wang et al., 2025a,b). Although individual ANS relays and pathways have been well-characterized (Wang et al., 2025a,b), accumulating evidence points to more direct crosstalk between the sympathetic and parasympathetic branches, which frequently act in coordination as a fundamental characteristic (Wang et al., 2025a,b).

Autonomic regulation is now understood to arise from multiple interactions between sympathetic and parasympathetic pathways at both central and peripheral levels (Ondicova and Mravec, 2010). Central interactions are primarily mediated by neurons within the paraventricular hypothalamic nucleus (PVN) and the nucleus of the solitary tract (NTS) (Ondicova and Mravec, 2010). Peripheral interactions arise from the structural and functional organization of sympathetic and parasympathetic pathways at sympathetic prevertebral ganglia (Wang et al., 2025a,b) and neuroeffector junctions (Ondicova and Mravec, 2010). A key example is the innervation of sympathetic prevertebral ganglia (notably the celiac ganglion) by vagal preganglionic neurons (Wang et al., 2025a,b; Ondicova and Mravec, 2010). Specifically, vagal fibers—primarily via the celiac branch (Kaestner et al., 2019)—project to and terminate within the celiac ganglion (Berthoud and Powley, 1996), which houses postganglionic sympathetic neurons regulating gastric functions (Wang et al., 2025a,b). Functionally, the celiac ganglion integrates inputs from spinal sympathetic preganglionic neurons, vagal parasympathetic preganglionic neurons, and visceral afferents (Kaestner et al., 2019). This convergence establishes the ganglion as a critical site for sympathovagal interactions (Ondicova and Mravec, 2010). Collectively, these intricate central and peripheral pathways may constitute a substrate for dysregulated sympathovagal interplay to precipitate gastric mucosal injury following SAH.

This study has several strengths. First, it addresses the long-standing controversy over vagal versus sympathetic dominance in SAH-induced gastric injury by simultaneously recording both autonomic nerves alongside tissue neurotransmitter measurements and selective interventions. Second, the multimodal approach—combining time-course pathology, direct nerve recordings, neurotransmitter assays, and surgical/pharmacological denervation—provides convergent evidence for a dysregulated sympathovagal interplay with potential predominant vagal involvement. Third, the 6- to 72-h temporal characterization delineates the onset, peak, and partial recovery of gastric mucosal injury after SAH. Together, these findings offer a novel framework for understanding the autonomic basis of stress-related mucosal disease in acute brain injury.

Several limitations of this study should be acknowledged. First, procedure-related mortality and animal exclusion (SAH score <8) reduced sample sizes in some subgroups, and several comparisons showed marginal significance, making the findings exploratory and necessitating replication in larger cohorts. Second, sham controls were included only at 6, 24, and 72 h (not at 12 and 48 h), as sham animals showed no time-dependent mucosal changes; this ethics-driven reduction limits direct time-matched comparisons with SAH groups for autonomic and neurotransmitter measures. Third, pharmacological blockade showed only trend-level histopathological reductions, and no dose-ranging study was conducted, so these findings are supportive rather than definitive, and dose-optimization studies are warranted. Fourth, the study does not explore central nuclei (e.g., paraventricular nucleus [PVN], dorsal motor nucleus of the vagus [DMV], rostral ventrolateral medulla [RVLM]) or underlying mechanisms (e.g., inflammation, oxidative stress, neurotransmitter imbalance) linking SAH to autonomic dysregulation, nor does it address peripheral mechanisms such as altered gastric perfusion, acid secretion, motility changes, or ischemia–reperfusion injury. Finally, the vagus nerve is a mixed nerve containing both afferent and efferent fibers, and no selective transection or pharmacological blockade was performed to distinguish their relative contributions to the recorded signals. Future studies incorporating larger sample sizes, selective nerve interventions, dose-optimized pharmacological blockade, and comprehensive mechanistic profiling are warranted to validate and extend these observations.

## 5 Conclusion

In summary, this study delineates the autonomic mechanisms underlying SAH-induced gastric mucosal injury. The mucosal injury, characterized by erosions and ulcerations, developed within 6 h post-SAH and peaked at 24 h, and was accompanied by concurrent activation of both gastric vagal and sympathetic nerves. Surgical blockade of either autonomic pathway attenuated gastric mucosal injury, with vagotomy demonstrating a more pronounced protective effect on macroscopic injury. At the tissue level, vagotomy significantly reduced both acetylcholine and norepinephrine. Collectively, these findings suggest that gastric pathogenesis after SAH involves dysregulated sympathovagal interplay, with a potential predominant role of vagal activity. However, given the exploratory nature of this study, further investigations with larger sample sizes and more comprehensive elucidation of the central and peripheral mechanisms that link SAH to autonomic dysregulation and consequent gastric mucosal injury are needed.

## Data Availability

The original contributions presented in the study are included in the article/supplementary material, further inquiries can be directed to the corresponding author.

## References

[ref1] Alhazzani W. Alshahrani M. Moayyedi P. Jaeschke R. (2012). Stress ulcer prophylaxis in critically ill patients: review of the evidence. Pol. Arch. Med. Wewn. 122, 107–114. doi: 10.20452/pamw.1173, 22354363

[ref2] Ali D. Barra M. E. Blunck J. Brophy G. M. Brown C. S. Caylor M. . (2021). Stress-related gastrointestinal bleeding in patients with aneurysmal subarachnoid hemorrhage: a multicenter retrospective observational study. Neurocrit. Care. 35, 39–45. doi: 10.1007/s12028-020-01137-5, 33150575

[ref3] Bansal V. Ryu S. Y. Lopez N. Allexan S. Krzyzaniak M. Eliceiri B. . (2012). Vagal stimulation modulates inflammation through a ghrelin mediated mechanism in traumatic brain injury. Inflammation 35, 214–220. doi: 10.1007/s10753-011-9307-7, 21360048 PMC3282000

[ref4] Barkun A. Bardou M. (2018). Proton-pump inhibitor prophylaxis in the Icu - benefits worth the risks? N. Engl. J. Med. 379, 2263–2264. doi: 10.1056/NEJMe1810021, 30354949

[ref5] Barletta J. F. Bruno J. J. Buckley M. S. Cook D. J. (2016). Stress ulcer prophylaxis. Crit. Care Med. 44, 1395–1405. doi: 10.1097/CCM.0000000000001872, 27163192

[ref6] Barletta J. F. Mangram A. J. Sucher J. F. Zach V. (2018). Stress ulcer prophylaxis in Neurocritical care. Neurocrit. Care. 29, 344–357. doi: 10.1007/s12028-017-0447-y, 28929324

[ref7] Berthoud H. R. Powley T. L. (1996). Interaction between parasympathetic and sympathetic nerves in prevertebral ganglia: morphological evidence for vagal efferent innervation of ganglion cells in the rat. Microsc. Res. Tech. 35, 80–86. doi: 10.1002/(SICI)1097-0029(19960901)35:1<80::AID-JEMT7>3.0.CO;2-W, 8873061

[ref8] Cho C. H. Dai S. Ogle C. W. (1978). Some observations on the gastric effects of phentolamine in rats. Eur. J. Pharmacol. 48, 51–55. doi: 10.1016/0014-2999(78)90043-2, 639844

[ref9] Cook D. Guyatt G. (2018). Prophylaxis against upper gastrointestinal bleeding in hospitalized patients. N. Engl. J. Med. 378, 2506–2516. doi: 10.1056/NEJMra1605507, 29949497

[ref10] Esler M. (2011). The sympathetic nervous system through the ages: from Thomas Willis to resistant hypertension. Exp. Physiol. 96, 611–622. doi: 10.1113/expphysiol.2010.052332, 21551268

[ref11] Esler M. Kaye D. (2000). Measurement of sympathetic nervous system activity in heart failure: the role of norepinephrine kinetics. Heart Fail. Rev. 5, 17–25. doi: 10.1023/A:1009889922985, 16228913

[ref12] Esterov D. Greenwald B. D. (2017). Autonomic dysfunction after mild traumatic brain injury. Brain Sci. 7:100. doi: 10.3390/brainsci7080100, 28800081 PMC5575620

[ref13] Evanson N. K. Veldhi P. Scherpenberg C. Riccobono J. M. Eid H. Mcguire J. L. (2025). Extracranial effects of traumatic brain injury: a narrative review. Clin. Pract. 15:47. doi: 10.3390/clinpract15030047, 40136583 PMC11941004

[ref14] Guth P. H. Aures D. Paulsen G. (1979). Topical aspirin plus Hcl gastric lesions in the rat. Cytoprotective effect of prostaglandin, cimetidine, and probanthine. Gastroenterology 76, 88–93. doi: 10.1016/S0016-5085(79)80133-X, 361495

[ref15] Harrison S. D. J. Bosin T. R. Maickel R. P. (1974). Physiological disposition of atropine in the rat. Pharmacol. Biochem. Behav. 2, 843–845. doi: 10.1016/0091-3057(74)90120-8, 4463377

[ref16] Hasegawa Y. Uchikawa H. Kajiwara S. Morioka M. (2022). Central sympathetic nerve activation in subarachnoid hemorrhage. J. Neurochem. 160, 34–50. doi: 10.1111/jnc.15511, 34525222

[ref17] Hayano J. Yuda E. (2019). Pitfalls of assessment of autonomic function by heart rate variability. J. Physiol. Anthropol. 38:3. doi: 10.1186/s40101-019-0193-2, 30867063 PMC6416928

[ref18] He Y. Qu Q. C. Wang B. X. Du F. Guo Z. H. (2013). Fos protein expression and role of the vagus nerve in the rat medullary visceral zone in multiple organ dysfunction syndrome caused by subarachnoid hemorrhage. Exp. Ther. Med. 5, 223–228. doi: 10.3892/etm.2012.770, 23251272 PMC3523947

[ref19] Hinson H. E. Sheth K. N. (2012). Manifestations of the hyperadrenergic state after acute brain injury. Curr. Opin. Crit. Care 18, 139–145. doi: 10.1097/MCC.0b013e3283513290, 22322258

[ref20] Kaestner C. L. Smith E. H. Peirce S. G. Hoover D. B. (2019). Immunohistochemical analysis of the mouse celiac ganglion: an integrative relay station of the peripheral nervous system. J. Comp. Neurol. 527, 2742–2760. doi: 10.1002/cne.24705, 31021409 PMC6722036

[ref21] Karádi O. Abdel-Salam O. M. Bódis B. Mózsik G. (1996). Preventive effect of atropine on indomethacin-induced gastrointestinal mucosal and vascular damage in rats. Pharmacology 52, 46–55. doi: 10.1159/000139360, 8966202

[ref22] Kemp W. J. Bashir A. Dababneh H. Cohen-Gadol A. A. (2015). Cushing's ulcer: further reflections. Asian J. Neurosurg. 10, 87–94. doi: 10.4103/1793-5482.154976, 25972936 PMC4421974

[ref23] Kerger B. D. James R. C. Roberts S. M. (1988). An assay for phentolamine using high performance liquid chromatography with electrochemical detection. Anal. Biochem. 170, 145–151. doi: 10.1016/0003-2697(88)90102-9, 3389507

[ref24] Khalid F. Yang G. L. Mcguire J. L. Robson M. J. Foreman B. Ngwenya L. B. . (2019). Autonomic dysfunction following traumatic brain injury: translational insights. Neurosurg. Focus 47:E8. doi: 10.3171/2019.8.FOCUS19517, 31675718

[ref25] Krishnamoorthy V. Komisarow J. M. Laskowitz D. T. Vavilala M. S. (2021). Multiorgan dysfunction after severe traumatic brain injury: epidemiology, mechanisms, and clinical management. Chest 160, 956–964. doi: 10.1016/j.chest.2021.01.016, 33460623 PMC8448997

[ref26] Kumaria A. Kirkman M. A. Scott R. A. Dow G. R. Leggate A. J. Macarthur D. C. . (2024). A reappraisal of the pathophysiology of Cushing ulcer: a narrative review. J. Neurosurg. Anesthesiol. 36, 211–217. doi: 10.1097/ANA.0000000000000918, 37188653

[ref27] Levinthal D. J. Strick P. L. (2020). Multiple areas of the cerebral cortex influence the stomach. Proc. Natl. Acad. Sci. USA 117, 13078–13083. doi: 10.1073/pnas.2002737117, 32434910 PMC7293610

[ref28] Liu Z. G. Gao J. L. Sun L. L. Guo X. Wang M. Pang Z. Y. . (2017). Preparation of a rat model of subarachnoid hemorrhage by a fiber core inserted in the internal carotid artery. Chin. J. Comp. Med. 27, 37–45. doi: 10.3969/j.issn.1671-7856.2017.06.008

[ref29] Liu B. Liu S. Yin A. Siddiqi J. (2015). Risks and benefits of stress ulcer prophylaxis in adult neurocritical care patients: a systematic review and meta-analysis of randomized controlled trials. Crit. Care 19:409. doi: 10.1186/s13054-015-1107-2, 26577436 PMC4650140

[ref30] Marcin B. Katarzyna S. Ivan K. (2024). The role of beta-adrenoreceptors in postoperative ileus in rats. Naunyn Schmiedeberg's Arch. Pharmacol. 397, 4851–4857. doi: 10.1007/s00210-023-02918-3, 38157026 PMC11166813

[ref31] Moussouttas M. Lai E. W. Khoury J. Huynh T. T. Dombrowski K. Pacak K. (2012). Determinants of central sympathetic activation in spontaneous primary subarachnoid hemorrhage. Neurocrit. Care. 16, 381–388. doi: 10.1007/s12028-012-9673-5, 22311230

[ref32] Moussouttas M. Mearns E. Walters A. Decaro M. (2015). Plasma catecholamine profile of subarachnoid hemorrhage patients with neurogenic cardiomyopathy. Cerebrovasc. Dis. Extra 5, 57–67. doi: 10.1159/000431155, 26120322 PMC4478315

[ref33] Mrozek S. Gobin J. Constantin J. M. Fourcade O. Geeraerts T. (2020). Crosstalk between brain, lung and heart in critical care. Anaesth. Crit. Care Pain Med. 39, 519–530. doi: 10.1016/j.accpm.2020.06.016, 32659457

[ref34] Ondicova K. Mravec B. (2010). Multilevel interactions between the sympathetic and parasympathetic nervous systems: a minireview. Endocr. Regul. 44, 69–75. doi: 10.4149/endo_2010_02_69, 20429636

[ref35] Rabinstein A. A. (2020). Autonomic hyperactivity. Continuum 26, 138–153. doi: 10.1212/con.0000000000000811, 31996626

[ref36] Rachfalska N. Putowski Z. Krzych L. J. (2020). Distant organ damage in acute brain injury. Brain Sci. 10:1019. doi: 10.3390/brainsci10121019, 33371363 PMC7767338

[ref37] Ray A. Gulati K. Henke P. (2020). Stress gastric ulcers and Cytoprotective strategies: perspectives and trends. Curr. Pharm. Des. 26, 2982–2990. doi: 10.2174/1381612826666200521143203, 32436823

[ref38] Rizoli S. B. Jaja B. N. Di Battista A. P. Rhind S. G. Neto A. C. Da C. L. . (2017). Catecholamines as outcome markers in isolated traumatic brain injury: the coma-Tbi study. Crit. Care 21:37. doi: 10.1186/s13054-017-1620-6 28228155 PMC5322658

[ref39] Robba C. Bonatti G. Pelosi P. Citerio G. (2020). Extracranial complications after traumatic brain injury: targeting the brain and the body. Curr. Opin. Crit. Care 26, 137–146. doi: 10.1097/MCC.0000000000000707, 32004191

[ref40] Salem R. Vallee F. Depret F. Callebert J. Maurice J. P. Marty P. . (2014). Subarachnoid hemorrhage induces an early and reversible cardiac injury associated with catecholamine release: one-week follow-up study. Crit. Care 18:558. doi: 10.1186/s13054-014-0558-1, 25358417 PMC4245729

[ref41] Schiller M. Ben-Shaanan T. L. Rolls A. (2021). Neuronal regulation of immunity: why, how and where? Nat. Rev. Immunol. 21, 20–36. doi: 10.1038/s41577-020-0387-1, 32811994

[ref42] Schirmer C. M. Kornbluth J. Heilman C. B. Bhardwaj A. (2012). Gastrointestinal prophylaxis in neurocritical care. Neurocrit. Care. 16, 184–193. doi: 10.1007/s12028-011-9580-1, 21748505

[ref43] Silverman H. A. Stiegler A. Tsaava T. Newman J. Steinberg B. E. Masi E. B. . (2018). Standardization of methods to record vagus nerve activity in mice. Bioelectron. Med. 4:3. doi: 10.1186/s42234-018-0002-y 32232079 PMC7098227

[ref44] Sugawara T. Ayer R. Jadhav V. Zhang J. H. (2008). A new grading system evaluating bleeding scale in filament perforation subarachnoid hemorrhage rat model. J. Neurosci. Methods 167, 327–334. doi: 10.1016/j.jneumeth.2007.08.004, 17870179 PMC2259391

[ref45] Tao J. Campbell J. N. Tsai L. T. Wu C. Liberles S. D. Lowell B. B. (2021). Highly selective brain-to-gut communication via genetically defined vagus neurons. Neuron 109, 2106–2115.e4. doi: 10.1016/j.neuron.2021.05.004, 34077742 PMC8273126

[ref46] Wang T. Teng B. Yao D. R. Gao W. Oka Y. (2025a). Organ-specific sympathetic innervation defines visceral functions. Nature 637, 895–902. doi: 10.1038/s41586-024-08269-0, 39604732

[ref47] Wang T. Tufenkjian A. Ajijola O. A. Oka Y. (2025b). Molecular and functional diversity of the autonomic nervous system. Nat. Rev. Neurosci. 26, 607–622. doi: 10.1038/s41583-025-00941-2, 40610604

[ref48] Wehrwein E. A. Orer H. S. Barman S. M. (2016). Overview of the anatomy, physiology, and pharmacology of the autonomic nervous system. Compr. Physiol. 6, 1239–1278. doi: 10.1002/j.2040-4603.2016.tb00714.x, 27347892

[ref49] Wei J. Jiang R. Li L. Kang D. Gao G. You C. . (2019). Stress-related upper gastrointestinal bleeding in adult neurocritical care patients: a Chinese multicenter, retrospective study. Curr. Med. Res. Opin. 35, 181–187. doi: 10.1080/03007995.2018.1448261, 29499622

[ref50] Wongsripuemtet P. Ohnuma T. Minic Z. Vavilala M. S. Miller J. B. Laskowitz D. T. . (2025). Early autonomic dysfunction in traumatic brain injury: an article review on the impact on multiple organ dysfunction. J. Clin. Med. 14:557. doi: 10.3390/jcm14020557, 39860563 PMC11765831

[ref51] Woolfrey S. G. Palin K. J. Davis S. S. (1989). The effect of Miglycol 812 oil on the oral absorption of propranolol in the rat. J. Pharm. Pharmacol. 41, 579–581. doi: 10.1111/j.2042-7158.1989.tb06534.x, 2571709

[ref52] Xu Y. Jia J. Xie C. Wu Y. Tu W. (2018). Transient receptor potential Ankyrin 1 and substance P mediate the development of gastric mucosal lesions in a water immersion restraint stress rat model. Digestion 97, 228–239. doi: 10.1159/000484980, 29428952

[ref53] Ziaka M. Exadaktylos A. (2024). Pathophysiology of acute lung injury in patients with acute brain injury: the triple-hit hypothesis. Crit. Care 28:71. doi: 10.1186/s13054-024-04855-w, 38454447 PMC10918982

